# Supplementary material for: Iterative improvement in the automatic modular design of robot swarms
Source: PeerJ Comput Sci. 2020 Dec 7;6:e322. doi: 10.7717/peerj-cs.322 (PMC7924708; doi:10.7717/peerj-cs.322)
Supplement: Supplemental Information 3 [file peerj-cs-06-322-s003.zip › argos3/doc/api/standalone/a00640_source.html]

ARGoS: plugins/simulator/visualizations/qt-opengl/qtopengl\_joystick.h Source File


- Main Page
- Related Pages
- Namespaces
- Classes
- Files

- File List
- File Members

# plugins/simulator/visualizations/qt-opengl/qtopengl\_joystick.h

Go to the documentation of this file.

```
00001 
00010 #ifndef QT_OPENGL_JOYSTICK_H
00011 #define QT_OPENGL_JOYSTICK_H
00012 
00013 #include <QObject>
00014 #include <QMap>
00015 #include <QTime>
00016 #include <QTimer>
00017 #include <QStringList>
00018 #include <SDL/SDL.h>
00019 
00020 #define SDL_JOYSTICK_DEFAULT_EVENT_TIMEOUT         25
00021 #define SDL_JOYSTICK_DEFAULT_AUTOREPEAT_DELAY      250
00022 
00023 namespace argos {
00024 
00025    class CQTOpenGLJoystick : public QObject
00026    {
00027       Q_OBJECT
00028 
00029    public:
00030       QStringList joystickNames;
00031       SDL_Joystick *joystick;
00032       int numAxes;
00033       int numButtons;
00034       int numHats;
00035       int numTrackballs;
00036       int eventTimeout;
00037       int autoRepeatDelay;
00038       bool autoRepeat;
00039       QTimer joystickTimer;
00040       QMap<int, int> deadzones;
00041       QMap<int, int> sensitivities;
00042 
00043       CQTOpenGLJoystick(QObject *parent = 0,
00044                         int joystickEventTimeout = SDL_JOYSTICK_DEFAULT_EVENT_TIMEOUT,
00045                         bool doAutoRepeat = TRUE,
00046                         int autoRepeatDelay = SDL_JOYSTICK_DEFAULT_AUTOREPEAT_DELAY);
00047       ~CQTOpenGLJoystick();
00048       void open(int);
00049       void close();
00050       inline bool isOpen() const { return joystick != NULL; }
00051       int getAxisValue(int);
00052       inline bool connected() const { return !joystickNames.isEmpty(); }
00053 
00054    private:
00055       QMap<int, Sint16> axes;
00056       QMap<int, Uint8> buttons;
00057       QMap<int, Uint8> hats;
00058       QMap<int, QTime> axisRepeatTimers;
00059       QMap<int, QTime> buttonRepeatTimers;
00060       QMap<int, QTime> hatRepeatTimers;
00061 
00062    signals:
00063       void axisValueChanged(int axis, int value);
00064       void buttonValueChanged(int button, bool value);
00065       void hatValueChanged(int hat, int value);
00066       void trackballValueChanged(int trackball, int deltaX, int deltaY);
00067 
00068    public slots:
00069       void processEvents();
00070    };
00071 
00072 }
00073 
00074 #endif
```

---

Generated on 10 Jul 2018 for ARGoS by 
 1.6.1
